# Nanoparticle-based drug delivery systems: A promising approach for the treatment of liver fibrosis

**DOI:** 10.1016/j.ijpx.2025.100411

**Published:** 2025-09-29

**Authors:** Ya-Ning Chen, Meng-Qi Li, Hui-Juan Zhang, Na-Na Xu, Yu-Qian Xu, Wen-Xuan Liu, Ting-Ting Chen, Nan Li, Guang-Yang Wu, Jie-Min Zhao, Wu-Yi Sun

**Affiliations:** a Institute of Clinical Pharmacology, School of Pharmacy, Anhui Medical University; Key Laboratory of Anti-inflammatory and Immune Medicine, Ministry of Education, Hefei 230032, China; b Department of Gastrointestinal Surgery, The Third Affiliated Hospital of Anhui Medical University (The First People's Hospital of Hefei), Hefei, Anhui Province 230061, China

**Keywords:** Nanocarriers, Drug delivery, Liver fibrosis

## Abstract

Liver fibrosis is the predominant pathological feature of chronic liver diseases, affecting the well-being of millions around the world. If not detected and intervened on time during the early stage, liver fibrosis can advance to cirrhosis, hepatic insufficiency, and finally hepatocellular carcinoma, thereby endangering human health seriously. Current pharmacotherapies for liver fibrosis have several limitations, such as a lack of sufficient therapeutic efficacy and the presence of adverse side effects. In light of these challenges, the use of nanoparticles (NPs) as drug delivery systems for liver fibrosis has gained significant traction, owing to their inherent characteristics, including safety, stability, controlled release, and targeted delivery. Compared to conventional dosage forms, nanomedicines exhibit distinct advantages, including enhanced bioavailability and targeted delivery of drugs. The employment of NP systems has quickly gained prominence as a viable strategy for the secure delivery of hepatoprotective nucleic acids and drugs in treating liver fibrosis. This comprehensive review examines the primary categories of NPs and elucidates the targeted mechanisms underlying NP-mediated drug delivery systems specifically designed for addressing liver fibrosis.

## Graphical Abstract

**Figure:**
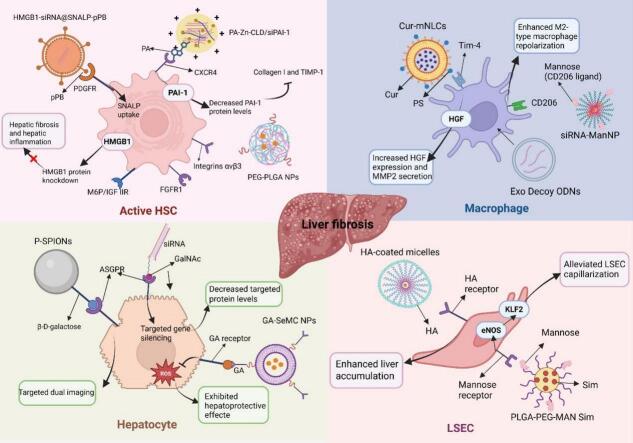

## 1 Introduction

In the past few decades, chronic liver diseases (CLDs) have increasingly become an escalating global disease burden and have become one of the main worldwide public health problems (Devarbhavi et al., 2023). CLDs have the potential to evolve into advanced liver fibrosis and eventually cirrhosis. Evidence demonstrates cirrhosis stands as the eleventh most common cause of mortality globally, causing roughly two million fatalities each year (Asrani et al., 2019). Given the high mortality associated with cirrhosis, there is an urgent need to emphasize the early detection and intervention of liver fibrosis (Man et al., 2023). Liver fibrosis results from the tissue repair response and is a common pathological feature of most CLDs, including viral, toxic, metabolic, and autoimmune diseases (Hammerich and Tacke, 2023; Henderson et al., 2020). The disease is mainly characterized by an over-accumulation of extracellular matrix (ECM) proteins due to a dysregulated response to wound and connective tissue repair (Parola and Pinzani, 2019). As damage persists and ECM accumulates, the liver gradually becomes more rigid and its function declines. Without timely detection and intervention, liver fibrosis has the potential to develop into cirrhosis, hepatocellular carcinoma, and other end-stage liver disorders (Zhang et al., 2016a). The development of liver fibrosis is driven by a sophisticated interplay between various cellular components within the liver, including hepatocytes, hepatic stellate cells (HSCs), Kupffer cells (KCs), and liver sinusoidal endothelial cells (LSECs). Among these, HSCs are considered to be the primary effector cells responsible for ECM deposition. When activated by inflammatory cytokines and oxidative stress, HSCs transdifferentiate into myofibroblasts, resulting in the overproduction of collagen and other ECM components. Consequently, targeting HSCs and other key cells has emerged as a potential approach for anti-fibrotic treatment (Ezhilarasan et al., 2018).

Current treatment strategies for liver fibrosis include etiological and anti-fibrotic treatment. In the early stages, it focuses on etiological treatment and protection against hepatic inflammation. When the disease progresses and leads to severe liver fibrosis, the implementation of anti-fibrotic treatment becomes imperative. Currently, there is no efficacious treatment beyond liver transplantation, the serious complications of which pose significant health threats to patients and create substantial medical burdens. Conventional pharmaceutical interventions for liver fibrosis frequently suffer from a lack of targeted efficacy, present significant adverse reactions, and demonstrate suboptimal efficacy (Shan et al., 2022). Consequently, there is an urgent requirement to formulate therapeutic approaches that are both effective and capable of targeted delivery for liver fibrosis.

With the ongoing emergence of NP-based drug delivery systems, there is a promising prospect of accurately delivering drugs directly to the liver. These systems, which vary in their structure, shape, size, and surface characteristics, significantly reduce adverse reactions and improve the therapeutic effect (Blanco et al., 2015). The treatment based on NPs has many advantages over conventional treatment. NPs can enhance the stability and solubility of encapsulated cargo. Moreover, they can cross biological barriers, facilitate transmembrane transport, prolong circulation times, and reduce adverse reactions (Mitchell et al., 2021). Despite the numerous advantages offered by NPs, factors such as altered hemodynamics and variations in ECM mechanical properties make it difficult for NPs to reach fibrotic liver tissue. Given that 30 %–99 % of administered NPs are naturally accumulated and retained in the liver after systemic delivery, many nanoparticulate systems have been developed for liver fibrosis (Zhang et al., 2016b). Among these, inorganic NPs, lipid NPs, polymeric NPs, and biomimetic NPs have drawn the interest of researchers due to their distinctive characteristics in drug transport. These NPs are recognized and processed by diverse liver cell types. Hepatocytes play a crucial role in the uptake and decomposition of NPs. In contrast, KCs are involved in the engulfment and clearance of these NPs. Moreover, HSCs become activated upon liver damage induced by NPs and engage in the subsequent repair processes. LSECs promote the material exchange between NPs and liver cells, thereby influencing the biological fate of NPs (Liu et al., 2025b). The review aims to systematically elaborate on various types of NPs and the applications of NP-based drug delivery systems to liver fibrosis.

## 2 Liver fibrosis

### 2.1 Pathogenesis of liver fibrosis

Liver fibrosis is an outcome of a disrupted tissue repair response following various forms of tissue damage, particularly during chronic inflammatory disorders. This condition develops after years or decades of continuous or recurrent organ damage accompanied by a persistent inflammatory reaction. It is distinguished by excessive accumulation and compositional changes in the ECM, particularly involving collagen deposition and the infiltration of fibrillar proteins like elastin into the space of Disse, leading to progressive architectural distortion of liver tissue.

**Fig. 1:**
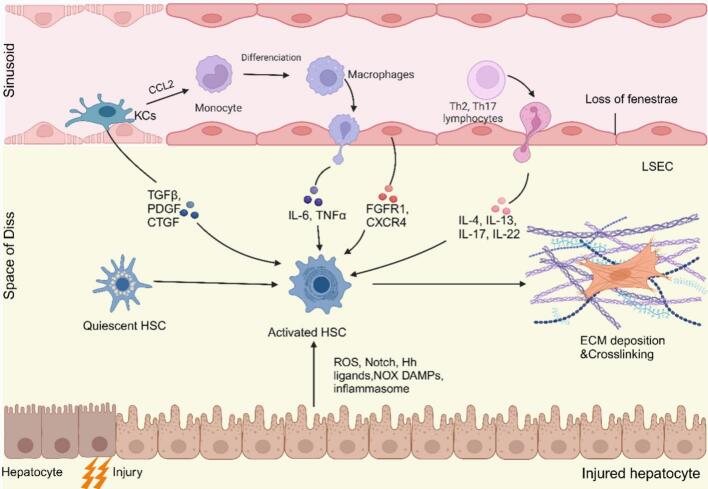
The mechanisms of liver fibrosis. Briefly, upon liver injury, hepatocytes undergo damage, releasing DAMPs, exosomes, *etc.* This will recruit immune cells to the injured region and secrete a myriad of inflammatory factors and chemokines, such as TNFα and TGFβ, which will stimulate the activation of quiescent HSCs. Once activated, HSCs produce a range of ECM components. Concurrently, LSECs undergo capillarization and accumulation of basement membranes. They can promote either liver regeneration or fibrosis. Abbreviations: CCL2, chemoattractant cytokine ligand 2; CXCR, C-X-C receptor; DAMPs, damage-associated molecular patterns; ECM, extracellular matrix; FGFR1, fibroblast growth factor receptor 1; GTGF, connective tissue growth factor; HSC, hepatic stellate cells; IL, interleukin; KCs, Kupffer cells; LSEC, liver sinusoidal endothelial cell; PDGF, platelet-derived growth factor; ROS, reactive oxygen species; TGFβ, transforming growth factor β; TNFα, tumor necrosis factor α; Th, helper T cell.

The fundamental mechanisms underlying hepatic inflammation have been recognized for many years. Persistent liver injury and the apoptosis of hepatocytes set off inflammatory responses. As a result, immune cells are called to the damaged region and emit a myriad of inflammatory factors and chemokines, including tumor necrosis factor α (TNFα) and transforming growth factor β (TGFβ). These substances stimulate the quiescent HSCs activation. Upon exposure to profibrotic stimuli, HSCs undergo a transdifferentiation process into myofibroblasts, resulting in the loss of vitamin A. Concurrently, they exhibit an upregulation of α-smooth muscle actin (α-SMA) and collagen production, along with matrix degradation inhibitors. This physiological response involves the replacement of normal parenchyma with connective tissue, a mechanism that serves to preserve the integrity and functionality of the organ. Nevertheless, the activation of HSCs also prompts the generation of pro-inflammatory cytokines and chemokines. These substances attract immune cells to the damaged liver, thus perpetuating the state of inflammation (Kisseleva and Brenner, 2021). The crosstalk between HSCs, damaged hepatocytes, macrophages, and other cell types contributes significantly to the mechanisms of liver fibrosis **(**Fig. 1**)**.

### 2.2 Cellular mechanism of liver fibrosis

The liver is primarily composed of two distinct cell types: parenchymal cells and non-parenchymal cells (Lotto et al., 2020). Among these, parenchymal hepatocytes are the predominant cell type, constituting 60 % of the total cellular content and accounting for 80 % of the overall liver volume. Liver fibrosis fundamentally originates from the replacement of damaged hepatocytes with myofibroblasts. Non-parenchymal cells such as HSCs, KCs, and LSECs are of crucial significance (Kumar et al., 2021). Liver fibrosis is typically triggered by alterations in these liver cells, therefore, therapeutic interventions for liver fibrosis primarily target HSCs, hepatocytes, KCs, and LSECs (Liu et al., 2024).

#### 2.2.1 HSCs

In their resting state, HSCs reside in the perisinusoidal space of Disse, positioned between hepatocytes and LSECs. Under normal physiological conditions, these HSCs remain in a dormant phase and store vitamin A within lipid droplets. However, upon liver injury induced by infections or hepatic toxins, HSCs receive activation signals from impaired hepatocytes and immune cells. This triggers their transdifferentiation into activated HSCs, which subsequently deposit substantial amounts of collagen and ECM molecules. As a result, they play a significant part in the advancement of liver fibrosis (Kamm and McCommis, 2022; Yin et al., 2013). Activated HSCs typically exhibit a loss of lipid droplets, an increase in α-SMA expression, and the tendency to migrate to the injury sites, facilitating liver repair. As the condition progresses, the abnormal buildup of ECM components and matrix metalloproteinases drives extensive tissue remodeling within the liver. This pathological process eventually leads to the substitution of functional hepatic tissue with non-functional fibrous scar tissue. A multitude of signals contribute to the activation of HSCs. The primary pathways leading to fibrosis, characterized by proliferation and fibrogenesis, encompass TGFβ, vascular endothelial growth factor, platelet-derived growth factor (PDGF), *etc.* Furthermore, multiple signaling pathways contribute to HSCs activation, including hedgehog signaling cascades, innate immune pathways (particularly Toll-like receptors and cytokine networks), G protein-coupled receptor signaling, and reactive oxygen species (ROS)-mediated mechanisms (Gu et al., 2021). The HSCs activation is extensively acknowledged as a crucial step in the development of liver fibrosis. As such, it serves as a key target for therapeutic strategies designed to alleviate this fibrotic condition.

#### 2.2.2 Hepatocytes

Hepatocytes play a crucial role in initiating inflammatory and fibrotic responses following hepatic injury. A growing array of mediators, such as ROS, Notch, Hh ligands, and NADPH oxidase (NOX), contribute to this process. Additionally, exosomes loaded with microRNAs released by injured hepatocytes have the potential to activate HSCs. Damage-associated molecular patterns (DAMPs) secreted from damaged hepatocytes also contribute to HSCs activation, either directly or indirectly, thereby promoting liver fibrosis progression. Additionally, high-mobility group box 1 (HMGB1), a nuclear protein released by compromised hepatocytes, directly activates HSCs. Consequently, strategies aimed at reducing hepatocyte injury and apoptosis represent a promising approach for preventing and treating liver fibrosis.

#### 2.2.3 Macrophages

Macrophages, the predominant immune cells in the liver, primarily consist of KCs (resident macrophages) and monocyte-derived macrophages (MoMφs). They play a crucial part in both the onset and progression of liver fibrosis (Wang et al., 2021; Wynn et al., 2013). KCs function as intravascular sentinels, detecting signals of cellular stress and damage originating from hepatocytes or external sources. They engage in the ingestion of cellular debris and have the capacity to activate inflammatory responses (Guilliams and Scott, 2022). In the initial phase of liver injury, KCs release CCL2, which serves to attract proinflammatory and profibrogenic MoMφs. Subsequently, KCs directly stimulate HSCs through the action of growth factors, including TGFβ and PDGF. Finally, KCs secrete proinflammatory cytokines and chemokines such as TNFα, IL-6, IL-1β, and CCL5. These interact with HSCs, thereby establishing a profibrogenic microenvironment (Wen et al., 2021). Consequently, targeting macrophages represents a promising therapeutic approach for addressing liver fibrosis.

#### 2.2.4 Other immune cells

In addition to macrophages, various other immune cell types invade the liver during fibrotic processes. Neutrophils, alternatively referred to as polymorphonuclear leukocytes or neutrophilic granulocytes, represent a key component of the circulating innate immune system. They migrate to areas of infection or injury, where they degrade damaged vessels and facilitate vascular growth. While neutrophils do not consistently contribute to hepatic inflammation in a functional manner, they can exacerbate liver disease. This process is accomplished through the secretion of cytotoxic reactive oxygen and nitrogen compounds, along with the production of pro-inflammatory mediators (Heymann and Tacke, 2016). Lymphocytes, essential components of adaptive immunity, regulate disease progression in liver injury. Both B and T cells simultaneously mediate and modulate inflammatory responses during antigen-driven conditions, including viral hepatitis and autoimmune liver disorders (Horn and Tacke, 2024). In the context of liver fibrosis, the immune response is predominantly characterized by the activation of Th2 and Th17 helper cells. These cell types play a significant role in driving the secretion of cytokines, including IL-4, IL-13, IL-17, and IL-22, which contribute to the progression of the fibrotic process. These cytokines critically activate HSCs and stimulate the release of ECM components (Reißing et al., 2024).

#### 2.2.5 LSECs

The vascular endothelium is composed of endothelial cells, which line the interior surfaces of arteries, veins, and capillaries, establishing a protective boundary between blood and surrounding tissues. These cells are vital for maintaining essential physiological processes, including ensuring blood remains fluid, regulating the migration of blood cells, and modulating responses of both innate and adaptive immunity (Krüger-Genge et al., 2019). LSECs, which are the most prevalent non-parenchymal cells in the liver, form the wall of the hepatic blood sinuses. In normal physiological states, these cells display anti-inflammatory and anti-fibrogenic properties achieved by inhibiting the activation of KCs and HSCs and regulating intrahepatic vascular resistance and portal pressure. However, when chronic liver injury occurs in the body, LSECs lose their fenestrae and undergo capillarization. These modified LSECs then secrete inflammatory mediators, attracting immune cells and worsening liver injury and inflammation (Hammoutene and Rautou, 2019). The LSEC response to liver injury could be the determining factor in whether the outcome is liver regeneration or fibrosis. Particularly, after an injury, the activation of the Chemokine (C-X-C motif) receptor (CXCR)7-ID1 pathway in LSECs tends to facilitate liver regeneration. Conversely, the fibroblast growth factor receptor (FGFR)1-CXCR4 pathway stimulates HSCs activation and contributes to fibrosis (Ding et al., 2014). Therefore, targeting LSECs is also one of the effective strategies for liver fibrosis.

### 2.3 Pharmacotherapy for liver fibrosis

The management of liver fibrosis encompasses both etiological and anti-fibrotic treatments. In the initial phases, emphasis is placed on etiological interventions and protective measures against hepatic inflammation. As the disease advances and significant liver fibrosis or cirrhosis develops, anti-fibrotic therapeutic strategies become imperative. Currently, the clinical options for targeted treatments of liver fibrosis and its early cirrhotic stages remain limited. At present, no efficacious treatment for liver fibrosis exists beyond liver transplantation. However, this procedure is accompanied by significant health risks and financial burdens for patients. Commonly used pharmaceuticals for liver fibrosis, including immunosuppressants, glucocorticoids, and anti-inflammatory drugs, often lack targeting capability, exhibit significant adverse reactions, and demonstrate suboptimal efficacy. For example, while sorafenib suggests a potential anti-liver fibrosis effect, its water insolubility significantly compromises its bioavailability, thereby impacting its pharmacodynamic efficacy (Ma et al., 2017). Moreover, it exhibits considerable toxicity and side effects in clinical applications, adversely affecting patients' quality of life and thus limiting its therapeutic utility. Despite the sustained endeavors in the clinical development of pharmaceuticals for liver fibrosis, no pharmaceutical formulations have been approved specifically for this condition and are available on the market. This may be attributed to the complex pathological mechanisms underlying liver fibrosis. Furthermore, there exists a significant disparity between the pathological conditions observed in animal models and those experienced by human patients, a factor that contributes to the limited efficacy of drugs during clinical trials (Guo and Lu, 2020). Additionally, pronounced adverse reactions elicited by large dosages constitute a primary cause for concern (Tan et al., 2021). For many decades, both physicians and researchers have worked to develop drug carriers that enhance liver-targeted delivery while reducing the adverse effects linked to drug metabolism.

The NP-based drug delivery system has gained significant attention as an innovative approach for targeted hepatic therapy, enabling precise drug delivery to the liver, minimizing adverse effects, and enhancing therapeutic outcomes. Current antifibrotic nanodrug therapies primarily target HSCs, hepatocytes, macrophages, LSECs, and other key cell types. To target HSCs, NPs equipped with PDGF receptor β (PDGFRβ)-targeting antibodies can be used, as they bind specifically to the HSCs surface. The asialoglycoprotein receptor (ASGPR) is uniquely found on hepatocytes and has a strong binding capability for ligands such as galactose or *N*-acetylgalactosamine ligands, which can be used to specifically target these cells. Similarly, NPs incorporating phosphatidylserine (PS) lipids can effectively target KCs and other macrophages. For specific targeting of LSECs, hyaluronic acid (HA)-based NPs can be employed, which is due to the high affinity of the HARE/Stabilin-2 receptor for HA.

## 3 The types and features of NPs

**Fig. 2:**
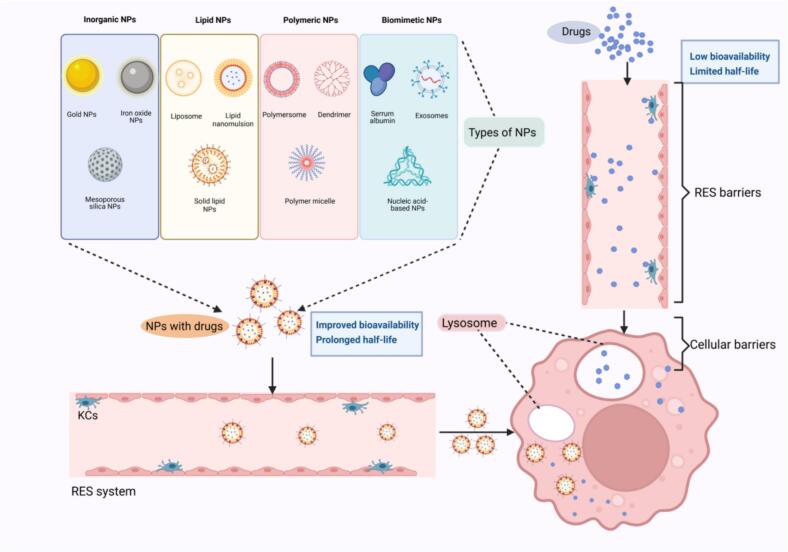
The types and features of NPs. NPs mainly include four types, inorganic NPs, lipid NPs, polymeric NPs, and biomimetic NPs. Additionally, NPs can improve the half-life of drugs and their ability to cross the RES and cellular barriers. Abbreviations: NP, nanoparticles; RES, reticuloendothelial system.

According to the International Organization for Standardization, NPs are defined as nano-objects with external dimensions predominantly in the nanoscale. More precisely, the lengths of the longest and shortest axes of the nano-object should not vary significantly. They can exhibit a wide variety of sizes, shapes, and structures. Moreover, NPs feature multiple layers, a surface layer often made of metal ions, small molecules, surfactants, or polymers, a shell layer constructed from a material that has different chemical properties compared to the core, and a core layer that serves as the central framework of the NPs (Joudeh and Linke, 2022). NPs are primarily categorized into two broad classifications: inorganic and organic, based on their chemical compositions. These categories can be further subdivided into metal oxide NPs, metal NPs, lipid NPs, polymeric NPs, and biomimetic NPs **(**Fig. 2**)**. The primary advantages of nanocarriers include excellent biocompatibility, enhanced bioavailability, minimized side effects, controlled release, targeted activity, and reduced systemic toxicity. Despite significant advancements in NP-based drug delivery systems, several critical challenges remain unresolved. One major issue is the incomplete understanding of the biodegradation process of NPs. Additionally, the *in vivo* kinetics of NPs, including their biodistribution, cellular uptake, and clearance pathways, require further investigation. The classes and characteristics of different nanoparticulate systems are discussed in Table 1.

**Table 1:** Features of different NPs.

Types of NPs		Advantages	Disadvantages
Inorganic NPs	Gold NPs	− High targeting efficiency − High stability and simple synthesis − Multiple sizes and shapes − Unique optical, electrical, and magnetic properties	− Limited biocompatibility and low solubility − Prone to accumulating in tissue and toxicity potential − Unsatisfactory clinic treatment outcomes − Regulatory hurdles
	Mesoporous silica	− Easy functionalization and surface modification − High capacity for storing drugs − Simple production process	− High costs
Lipid NPs		− High biocompatibility − Enhanced drug loading capacity − Wide adaptability to drugs − Reduced adverse effects, avoiding bio-clearance of agents − Clinically validated platform	− Moderate targeting efficiency and limited active targeting − Low systemic toxicity and potential phospholipid-induced immunogenicity − Rapid clearance by RES − High manufacturing costs
Polymeric NPs	Natural polymers	− High biocompatibility and degradability − High targeting efficiency − Controlled release kinetics − Scalable production	− Minimal hepatotoxicity and rare inflammatory responses − Potential polymer degradation products − and low stability − High manufacturing costs
	Synthetic polymers	− High bioavailability, stability, and loading capacity	− Low degradability and unfavorable side effects
Biomimetic NPs		− Excellent biocompatibility − High targeting efficiency − Low immunogenicity and high safety	− Inherent instability and lack of unified biological assessment − Standardization challenges − Difficulty in production, purification, and characterization

Abbreviations: NPs, nanoparticles. RES, reticuloendothelial system

### 3.1 Inorganic NPs

Inorganic NPs are distinguished by their metallic oxide or metallic core, typically encapsulated by a protective layer. The distinctive optical, electrical, and magnetic characteristics of these NPs depend on their sizes and shapes, a characteristic that organic NPs cannot replicate (Wang et al., 2022; Woo et al., 2023). For instance, Hirn et al. (Hirn et al., 2011) conducted a study on Wistar-Kyoto rats using AuNPs of varying sizes and surface charges, ranging from 1.4 to 200 nm, to investigate their biodistribution *via* intravenous administration. The results indicated that 50 % of 1.4 nm AuNPs and over 99 % of 200 nm AuNPs accumulated in the liver 24 h post-injection. Smaller gold NPs can have a longer circulation time in the blood due to their small size, which allows them to pass through the fenestrations in the liver sinusoidal endothelium more easily. However, they are still subject to phagocytosis by KCs. Larger gold NPs are more likely to be physically entrapped in the liver sinusoids and are also more readily recognized and phagocytosed by KCs.

Furthermore, the ability to modify their shapes is a significant feature of inorganic NPs. Substances like gold, iron, and silica are commonly utilized to create nanostructured systems tailored for diverse drug delivery applications. Gold NPs (AuNPs) are utilized in diverse forms, such as nanospheres, nanorods, nanoshells, and nanocages (Singh et al., 2018). Gold nanorods (NRs) have emerged as one of the emerging materials in recent years (Zheng et al., 2021). Mesoporous silica NPs have also been effectively employed for drug delivery due to their distinctive mesoporous structure, advantageous chemical properties, and biocompatibility (Xu et al., 2019). Studies have shown that mesoporous silica NPs loaded with salvianolic acid B (SAB) significantly reduce ROS levels and inhibit the proliferation of LX-2 cells more effectively than free SAB, showcasing a more potent antifibrotic impact (He et al., 2010).

While most inorganic materials have good stability, their clinical utility is constrained due to poor solubility and possible toxicity, especially in formulations that make use of heavy metals (Manshian et al., 2017). The inability of inorganic NPs to biodegrade is a significant limitation to their potential use in humans (Tee et al., 2019). Inorganic NPs typically exhibit a slow degradation rate. The body primarily clears these NPs *via* the reticulo-endothelial system (RES), which includes the liver and spleen. Larger inorganic NPs are more likely to be captured in the liver and spleen. Smaller inorganic NPs may remain in the bloodstream for a longer period, potentially being excreted through the kidneys. However, this process is limited, as the majority of small inorganic NPs are eventually assimilated by the RES. In addition, the surface properties of inorganic NPs significantly influence their clearance (Croissant et al., 2017). Neutral, hydrophilic NPs are less likely to be rapidly cleared by the RES compared to hydrophobic or charged NPs.

### 3.2 Lipid NPs

Unlike traditional liposomes, lipid NPs feature a unique micellar core structure, which can be modified by adjusting formulation and synthesis parameters (Leung et al., 2015). Lipid NPs contain four components. Cationic or ionizable lipids play two key roles: they bind to genetic material and assist in endosomal escape. Phospholipids are responsible for constructing the particle's structure. Cholesterol helps enhance the stability of the lipid NPs and promotes membrane fusion. As for pegylated lipids, they contribute to improving the stability of the particles and prolonging their circulation time (Hald et al., 2022; Schoenmaker et al., 2021). The size of lipid NPs plays a crucial role in their liver accumulation. Small NPs can have a longer circulation time and are more likely to enter the liver through the leaky blood vessels. Larger NPs are readily taken up by KCs. Additionally, chemical group modifications alter the hydrophilic membrane shell, enabling the liposome to target specific liver cell types (Paunovska et al., 2019). In the past decades, studies have found the transfection characteristics of lipoplexes comprised of positively charged cationic lipids and nucleic acid drugs. Concurrently, there has been significant progress in the development of lipid NPs using ionizable cationic lipids. These foundational studies have positioned lipid NPs as promising delivery vectors for nucleic acid therapeutics (Tenchov et al., 2021; Cullis and Felgner, 2024). RNA interference (RNAi) technology offers the ability to suppress the expression of pathogenic proteins, thereby offering a viable treatment for diseases caused by their activity. Since small interfering RNA (siRNA) is a polyvalent anionic and highly hydrophilic mid-size molecule, it exhibits limited cellular uptake. Furthermore, siRNA is prone to being degraded by nucleases in the bloodstream, resulting in poor accumulation in target tissues. Consequently, an efficient delivery system is required to transport siRNA drugs, which can help achieve gene silencing (Yonezawa et al., 2020). Notably, they facilitated the advent of the first siRNA drug, the development of COVID-19 mRNA vaccines, and advancements in gene editing methodologies (Van Der Meel et al., 2025). They are generally more biodegradable compared to inorganic NPs, depending on the composition of the phospholipids and the presence of specific enzymes. However, there are still some challenges that need to be addressed to expand their broader application. Low systemic toxicity, potential phospholipid-induced immunogenicity, and limited active targeting, though relatively well-documented, remain critical concerns. Additionally, the production and manufacturing processes pose significant obstacles, particularly when integrating NPs with complex therapeutic modalities (Cheng et al., 2024).

### 3.3 Polymeric NPs

Polymeric NPs are generally effective delivery systems because of their biocompatibility and straightforward formulation process. These NPs can encapsulate therapeutic agents within their core or entrap them in the polymer matrix. The structural characteristics of polymeric NPs play a crucial role in shielding drugs from serum-related loss and preventing opsonization by the complement system, which typically causes rapid drug clearance from circulation. Moreover, optimally designed polymeric NPs are anticipated to decrease the toxicity of therapeutic compounds (Hwang et al., 2020). Polymers include synthetic biodegradable types like poly(lactic acid) (PLA), polycaprolactone, poly (lactic-*co*-glycolic acid) (PLGA), and polyethylene glycol (PEG), as well as natural macromolecules such as chitosan, polysaccharides, gelatin, and starch. In the past few years, the use of polymeric NPs as delivery systems has significantly increased. Among the aforementioned biodegradable polymers, PLGA stands out due to its hydrolyzable ester links, which are approved by the FDA (Lv et al., 2022). In addition, PEG is frequently employed as a linker for the decorative placement of substrates or ligands on the NP surface (Kou et al., 2018). The PEG shell surrounding polymeric micelles minimizes protein adsorption, prolongs circulation time, and reduces non-specific uptake. The surface modification of PLGA NPs can also affect their liver accumulation. For example, NPs coated with targeting ligands may have a different accumulation pattern compared to non-targeted NPs. If the targeting ligand is specific to liver cells, it can enhance the uptake of PLGA NPs by these cells and increase their accumulation in the liver. In a research, Lin et al. (Lin et al., 2016) have formulated and optimized NP formulations by integrating PEG-PLGA copolymers with PLGA matrices. This innovative approach was specifically designed for the systemic delivery of sorafenib to hematic tissues, demonstrating the potential of such formulations in targeted therapeutic applications. However, polymeric NPs also have some disadvantages, such as difficulty in sterilization and the possibility of aggregation and toxicity.

The clearance of organic NPs is also influenced by their size and surface properties. Smaller organic NPs can be excreted through the kidneys, while larger ones are removed by the RES. Additionally, some organic NPs can be metabolized into non-toxic by-products and excreted through the respiratory or digestive systems. Generally, organic NPs exhibit lower immunogenicity compared to their inorganic counterparts. However, under specific conditions, they still have the potential to elicit immune responses. For instance, these organic NPs can stimulate the immune system if they are loaded with antigens or their surfaces are embellished with immunogenic molecules. Additionally, certain synthetic polymers utilized in the formulation of NPs might provoke mild immune reactions in sensitive individuals.

### 3.4 Biomimetic NPs

Biomimetic carriers offer a novel approach to drug delivery, leveraging the unique functions and superior delivery mechanisms of endogenous cells. This is attributed to its ability to evade detection by the mononuclear phagocyte system, which subsequently reduces immunogenicity and prolongs circulation time.

#### 3.4.1 HAS and BSA

Protein aggregates like human serum albumin (HAS) and bovine serum albumin (BSA) NPs are components of the body, and they will not be rejected by the immune system, with low immunogenicity and high safety characteristics (di Masi, 2023; Solanki et al., 2021). The secreted protein acidic and rich in cysteine (SPARC), alternatively referred to as BM-40, is a matricellular protein. It is significantly expressed by active HSCs once liver fibrosis occurs (Jiang et al., 2023). Luo et al. (Luo et al., 2023) developed nanoplatforms, specifically silibinin albumin nanocrystals (SLB-HSA NCs), to target active HSCs for treating liver fibrosis. The albumin coating on the nanocrystals enhanced their uptake by active HSCs *via* SPARC-mediated endocytosis. Additionally, pharmacokinetic studies revealed that the bioavailability of SLB-HSA NCs was notably higher than that of free SLB. After tail-vein injection, SLB-HSA NCs showed significant accumulation in fibrotic liver tissue and exhibited enhanced antifibrotic effects in mice with liver fibrosis. Lam et al. (Lam et al., 2015) employed glucose as a substitute for glutaraldehyde to modify albumin NPs for berberine delivery, thereby mitigating potential human toxicity.

#### 3.4.2 Exosomes and stem cells

Exosomes, small vesicles with a diameter ranging from 40 to 160 nm, are released from the plasma membrane through outward budding (Kalluri and LeBleu, 2020). As they are native to the human body rather than a foreign material, they can avoid an immune response, resulting in low immunogenicity. Furthermore, exosomes exhibit a pronounced capacity to target specific tissues or cells. They contain a variety of molecules that could be instrumental in treating diseases. Through genetic engineering, desirable ligands can also be incorporated onto their surfaces (Lee et al., 2023). Exosome targeting can achieve optimal therapeutic outcomes with lower drug concentrations, showcasing significant application value. Relative to alternative drug delivery mechanisms, such as liposomes, exosome-based drug delivery systems present superior stability, diminished toxicity, and precise targeted delivery (Liu et al., 2023). Despite significant advances, the field of this therapy still faces obstacles in terms of manufacturing, purification, and characterization. The process of preparing exosomes is hindered by high costs, low yields, and inconsistent methodologies. Consequently, there is a significant need to explore the preparation of exosomes from diverse species or cell sources to identify methods that are straightforward, consistent, and cost-effective. Mesenchymal stem cells (MSCs), which originate from mesenchymal and connective tissues such as bone marrow and liver, secrete cytokines and signaling molecules with the potential to suppress inflammatory responses (Lin et al., 2011). MSC-Exos have shown promise as drug delivery carriers (Lu et al., 2023). However, the precise mechanisms through which combination therapy mitigates liver fibrosis remain elusive, thus precluding its widespread clinical application. (Xu et al., 2024).

#### 3.4.3 Nucleic acid-based NPs-

In recent years, nucleic acid-based nanocarriers have gained significant attention as drug delivery systems due to their excellent biocompatibility and minimal toxicity. These carriers offer programmable structures with advantages such as controllable size and modifiability (Guo et al., 2022). DNA nanotechnology facilitates the creation of an array of 2D and 3D structures in various sizes and shapes, such as tetrahedra, octahedra (Oh et al., 2024), complicated DNA origami structures (Jiang et al., 2024), *etc.* Considering the analogous chemical properties of DNA and RNA, it is a viable strategy to bind therapeutic RNA to structural single-stranded DNA, enabling the loading of miRNA regulators onto nucleic acid-based nanocarriers. This approach holds promise for promoting the development of nucleic acid drugs (Li et al., 2024). During the siRNA delivery process, DNA nanostructures serve dual functions. They can not only shield the cargo from nuclease degradation but also read and respond to environmental changes. Among the various DNA nanostructures, the DNA tetrahedron stands out as a typical three-dimensional framework. It boasts remarkable assembly efficiency and structural rigidity, resulting in high stability and precise structural control. This makes it particularly promising for applications in gene regulation (Wei et al., 2023), drug delivery (Velusamy et al., 2024), cellular biosensors (Xie et al., 2017), and others. A notable example is the DNA tetrahedron, fabricated by Wei et al. (Wei et al., 2024), which was designed for drug delivery for liver disease treatment.

Despite their numerous advantages, potential, and high promise, DNA nanostructures face several challenges. The most prominent of these issues is the insufficient knowledge regarding pharmacokinetics, which encompasses *in vivo* circulation, distribution, metabolism, and excretion. Secondly, the cost of production is an issue. For viable biomedical applications, it is crucial to be capable of preparing functional DNA nanostructures at the potential gram scale. Thirdly, biosafety poses another issue; while DNA is biodegradable and biocompatible, these properties may change when it is engineered into a nanostructure. A comprehensive study of the immunostimulatory properties of DNA nanostructures is necessary before any clinical bio-applications can be considered (Hu et al., 2019).

Biomimetic NPs are engineered to emulate the structure and function of biological entities, and consequently, their degradation patterns closely resemble those of their natural counterparts. In addition, biomimetic NPs can leverage the body's inherent clearance mechanisms associated with the biological entities they emulate. Typically, biomimetic NPs are anticipated to exhibit minimal immunogenicity owing to their biomimetic design. Nevertheless, any deviations between the biomimetic NPs and their natural counterparts, or modifications with non-self molecules, might trigger immune reactions.

We also focus on novel approaches, such as stimuli-responsive NPs (SRNP), theranostics, artificial intelligence-optimized NPs (AI-NP), or organoid-based screening platforms. SRNP relies on microenvironment responses to release the payload. For example, bilirubin (BR) exhibits efficacy when incorporated into various NPs for cancer therapy. Moreover, BR-loaded NPs can be engineered to become stimuli-responsive carriers. These offer the potential for a sustained, controlled, and on-demand release of drugs in response to internal or external stimuli such as reactive oxygen species, glutathione, light, enzymes, and acidic pH (Mirhadi et al., 2024). In contrast, theranostic NPs, which combine diagnostic and therapeutic features in a single formulation, can provide information about their biodistribution and about target site accumulation, distribution, and retention (Pallares et al., 2022). AI-NP is developed through computational approaches focused on targeted delivery. Organoids, three-dimensional structures that accurately mimic tissue architecture and cellular composition, hold significant potential for organoid-based drug screening. Compared to the conventional manual evaluation method, the organoid size ratio method offers a more objective, rapid, and labor-efficient approach, making it ideal for high-throughput drug screening (Li et al., 2022a). With the development of nanotechnology, nanoformulations are expected to assist researchers in overcoming established barriers, ultimately implementing safe, effective, and patient-specific therapies.

## 4 NP-based drug delivery systems for liver fibrosis

The mechanisms for targeting drug delivery *via* NPs are primarily classified into passive targeting, active targeting, and endogenous organ targeting (Dilliard and Siegwart, 2023).

### 4.1 Passive targeting

Particularly, as the main passive targeting organ, the liver plays a critical role in the metabolism and clearance of NPs. In the absence of a targeting moiety, all NPs invariably accumulate in the liver. Passive strategies rely on the accumulation of NPs in the liver to increase local drug concentration while minimizing off-target delivery to other organs, thereby reducing adverse side effects. To achieve this, NPs are often tailored by modifying their size, enhancing surface properties to minimize non-specific interactions, or applying them locally. As a result, their passive targeting of specific sites relies on anatomical or pathological conditions that align with the characteristics of the NPs. The enhanced permeability and retention effect is a prevalent passive targeting strategy extensively employed in the treatment of solid tumors (Cheng et al., 2021), and its potential application for liver fibrosis is currently under investigation. Studies have demonstrated that particle sizes between 50 and 200 nm can escape from the splenic space, enter the sinusoidal endothelial pores, and accumulate in large quantities. Small neutral lipophilic particles, measuring less than 100 nm, can pass through the liver parenchyma and enter mesenchymal cells, whereas larger particles are rapidly phagocytosed by KCs (Ngo et al., 2022).

A multitude of studies have documented the propensity for inorganic NPs to accumulate in the liver in comparison to other organs, such as the spleen and lung. Tumor necrosis factor-stimulated gene 6 (TSG-6) displays potent antifibrotic activity and is capable of inducing M2 macrophage polarization (Li et al., 2019). In a study, Wang et al. (Wang et al., 2020) synthesized calcium phosphate nanoparticles (CaP NPs) using an approach with BSA as the bio-template. NPs encapsulating BSA, with sizes ranging from 100 to 200 nm, exhibit potential as an effective liver-targeted drug delivery system. Polymeric NPs, constructed from biodegradable polymers such as polycaprolactone, PLGA, undergo degradation *via* hydrolysis of their ester bonds, and their size and surface properties can be easily tuned. Smaller PLGA NPs can have a longer circulation time in the blood and may enter the liver through the leaky blood vessels in pathological conditions. Larger PLGA NPs are more likely to be phagocytosed by KCs. Compared to these NPs, biomimetic NPs might exhibit superior stealth characteristics, enabling them to circumvent swift removal by the RES.

Due to altered hemodynamics, ECM mechanical properties, and complex physiological processes in liver fibrosis, NPs encounter challenges in reaching fibrotic liver tissue. In a normal liver, the sinusoidal endothelial cells have fenestrations. However, during liver fibrosis, these fenestrations are gradually lost. The loss of fenestrations creates a physical barrier that restricts the access of NPs to hepatocytes and other target cells in the liver. Fibrotic liver is characterized by an overproduction of ECM, leading to a stroma-rich microenvironment. Additionally, activated HSCs and immune cells secrete cytokines and chemokines that further contribute to the complexity of the microenvironment. NPs may interact non-specifically with the ECM components, leading to their aggregation and clearance from the circulation. Moreover, the cytokines and chemokines in the microenvironment can affect the stability and targeting ability of NPs.

### 4.2 Active targeting

**Fig. 3:**
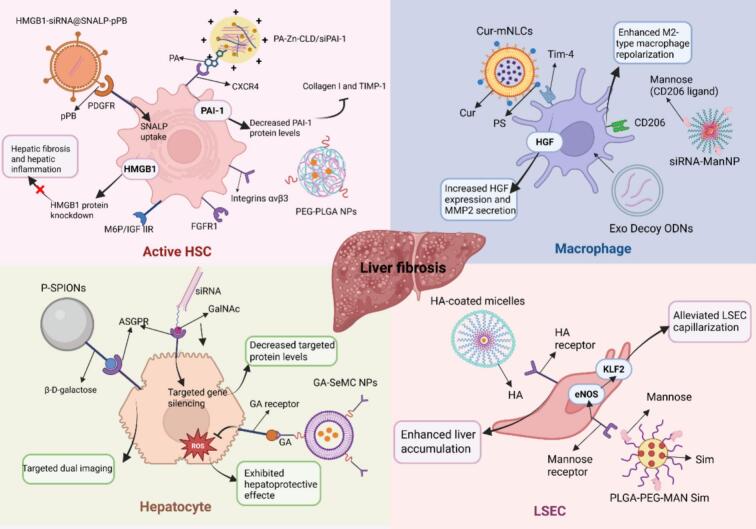
Active targeting of NP-based drug delivery systems for liver fibrosis. Liver fibrosis possesses multiple targeting cells, mainly including HSCs, hepatocytes, KCs, and LSECs. Lipid NP, polymeric NP, inorganic NP, and biomimetic NP-based drug delivery systems modified by receptor-specific ligands have been employed in liver fibrosis treatment. For instance, PDGFR, CXCR4, M6P/IGF IIR, *etc.*, which are abundantly expressed on HSCs surfaces, have been designed with corresponding recognition ligands to deliver antifibrotic drugs to HSCs. Abbreviations: ASGPR, asialoglycoprotein receptor; CD206, mannose receptor; CXCR, chemokine (C-X-C motif) receptor; Cur, curcumin; eNOS, endothelial nitric oxide synthase; Exos, exosome; FGFR1, fibroblast growth factor receptor 1; GA, glycyrrhetinic acids; GalNAc, *N*-acetylgalactosamine; HA, hyaluronic acid; HGF, hepatocyte growth factor; HSC, hepatic stellate cell; HMGB1, high-mobility group box 1; KLF2, kruppel-like factor 2; LSEC, liver sinusoidal endothelial cell; MAN, mannan; M6P/IGF IIR, mannose 6-phosphate/insulin-like growth factor II receptor; mNLCs, phosphatidylserine-modified nanostructured lipid carriers; NCs, nanocrystals; NP, nanoparticle; ODNs, oligodeoxynucleotides; PA, CXCR4 ligand; PAI-1, plasminogen activator inhibitor-1; PDGFR, platelet-derived growth factor receptor; PEG-PLGA, poly(ethylene gly*co*l)-blockpoly(lactide-co-glycolide); PLGA, poly (l-lactide-*co*-glycolide); pPB, a cyclic peptide that can act as a targeting ligand for PDGFRβ; PS, phosphatidylserine; P-SPIONs, pullulan-stabilized iron oxide nanoparticles; SeMC, L‑selenium-methylselenocysteine; siRNA, small interfering RNA; SNALP, stable nucleic acid lipid NPs; Tim-4, T-cell immunogloblin domain and mucin domain 4; TIMP-1, tissue inhibitor of metalloproteinases-1.

Active targeting strategies have high specificity, which can accurately identify and combine, thereby improving the enrichment of NPs at the target site. Different from passive targeting, active targeting employs surface decoration with cell-specific ligands like proteins, peptides, antibodies, or carbohydrates. These ligands then bind to the corresponding receptors at the cell membrane (Böttger et al., 2020). Active targeting technology offers the potential for precise delivery, thereby minimizing toxic side effects within the organ. This section provides a detailed summary of NP-based drug delivery systems designed to target specific cells of liver fibrosis **(**Fig. 3**)**. The main systems for liver fibrosis are detailed in Table 2.

**Table 2:** Active targeting NP-based drug delivery systems for liver fibrosis.

Targeting cell	Receptor	Ligand	NPs	Therapeutic drugs/methods	NP formulation	Animal model/Cell lines	Outcome	Reference
HSCs	PDGFR	PDGFRβ	GNR	PPTT	GNR-PDGFRβ	CCl_4_-induced mouse model/Immortalized normal primary mouse HSCs (GRX cells)	Reduced liver inflammation and enhanced the inhibition of HSCs activation	(Ribera et al., 2021a)
		pPB peptide (C*SRNLIDC*)	SNALP	HSP47 siRNA	pPB-SNALPs	TAA-induced mouse model/LX2 cells (Human HSCs) and the primary HSCs	Reduced the expression of gp46 mRNA and inhibited collagen deposition	(Jia et al., 2018)
			SNALP	HMGB1-siRNA	HMGB1-siRNA@SNALP-pPB	TAA- or CCl_4_-induced mouse model/Mouse HSCs	Inhibited the activation and proliferation of HSCs, reduced the release of HMGB1 protein, and inhibited collagen deposition	(Zhang et al., 2020)
	M6P/IGF IIR	IGF IIR-targeting peptide	SLN	MT	M6P-HSA-MT-SLN	BDL- or CCl_4_-induced mouse model	Reduced liver inflammatory infiltration and inhibited collagen deposition	(Tan et al., 2023)
	CXCR4	PA	PA-Zn-CLD nanocomplex	PAI-1 siRNA	PA-Zn-CLD/siPAI-1	HSC-T6 cells	Decreased PAI-1 protein levels and the expression of collagen I and TIMP-1	(Wu et al., 2022)
	CD44	hyaluronan	Self-assembled HA-BR	Bilirubin	HABNs	CD-HFD-induced mouse model/LX2 cells (Human HSCs)	Inhibited HSCs activation and proliferation, and collagen production.	(Shinn et al., 2024)
			Polymeric NPs	PRB, COL	HA@PRB/COL NPs	CCl_4_-induced mouse model/LX2 cells (Human HSCs)	Inhibited HSCs activation and proliferation, and reduced collagen I production	(Wang et al., 2024)
	Integrins αvβ3	RGD peptide (cRGDfK)	PEG-PLGA NPs	GMO, miR-29b	GMO- and miR-29b-loaded cRGD-PEG-PLGA NPs	HSCs cells/CCl_4_-induced mouse model	Enhanced inhibition of HSCs activation and collagen I production	(Ji et al., 2020)
	RBPR	RBP-vitamin A complex	LNPs	Col1α1 siRNA	siCol1α1 VLNPs	CCl_4_-induced mouse model	Increased both mRNA and protein expression levels of Col1α1 and inhibited collagen production	(Qiao et al., 2020)
	FGFR1	FGF2	SPIONs	FGF2	FGF2-SPIONs	CCl_4_-induced mouse model/TGFβ-activated LX2 cells (Human HSCs)	Enhanced inhibition of HSCs activation, migration, contraction, and collagen production	(Kurniawan et al., 2020)
Hepatocytes	ASGPR	GalNAc	SPIONs	PPTT	P-SPIONs	CCl_4_-induced mouse model/HepG2 cells	Facilitated targeted dual imaging in liver fibrosis	(Saraswathy et al., 2021)
	GA receptor	GA	NPs composed of amphiphilic polymers	SeMC	GA-SeMC NPs	APAP- or CCl_4_-induced mouse model/L02 (Normal liver cell line)	Suppressed the levels of liver oxidative stress, histological lesions, and serum transaminases, and promoted the expression of antioxidant enzymes	(Yuan et al., 2023)
Macrophages	Tim-4	PS	Lipid NPs	Cur	Cur-mNLCs	CCl_4_-induced mouse model	Increased HGF expression and MMP2 secretion	(Wang et al., 2018)
	CD206 (Mannose receptor)	CD206 ligand (Mannose)	Nanohydrogel particles	CSF-1R siRNA	CSF-1R siRNA-ManNP	CCl_4_-induced mouse model/THP-1 cells (Human macrophages)	Decreased CSF-1R protein levels and enhanced M2-type macrophage repolarization	(Kaps et al., 2020)
LSECs	Mannose receptor	Mannan	PLGA-PEG-MAN NPs	Sim	PLGA-PEG-MAN Sim	CCl_4_-induced mouse model/SK-Hep1 cells (LSEC lines)	Increased both mRNA and protein expression levels of KLF2 and eNOS, and alleviated LSEC capillarization	(Yu et al., 2022)

Abbreviations: APAP, paracetamol; ASGPR, asialoglycoprotein receptor; BDL, bile duct ligation; BMDMs, Bone Marrow Derived Macrophages; CCl_4_, carbon tetrachloride; CD-HFD, choline-deficient l-amino acid-defined high-fat diet; Chol, cholesterol; CLD, clodronate; COL, collagenase type I; cRGD, cyclic arginine-glycine-aspartic sequence; CSF-1R, colony-stimulating factor 1 receptor; C*SRNLIDC*, pPB peptide, a cyclic peptide can act as a targeting ligand for PDGFRβ; Cur, curcumin; CXCL: chemokine (C-X-C motif) ligand; CXCR, chemokine (C-X-C motif) receptor; ECM, extracellular matrix; eNOS, endothelial nitric oxide synthase; FGF2, fibroblast growth factor 2; GA, glycyrrhetinic acids; GalNAc, *N*-acetylgalactosamine; GMO, germacrone; GNR, gold nanorods; G4, G-quadruplex; HABNs, hyaluronic acid-bilirubin nanoparticles; HAS, human serum albumin; HGF, hepatocyte growth factor; HSA, human serum albumin; HSCs, hepatic stellate cells; HSP47, heat shock protein 47; HMGB1, high-mobility group box 1; KCs, Kupffer cells; KLF2, kruppel-like factor2; LSECs: liver sinusoidal endothelial cells; MAN, mannan. Mal, maleimide; MCDHF, methionine-choline-deficient and high-fat; mNLCs, phosphatidylserine-modified nanostructured lipid carriers; MMP2, matrix metalloproteinases 2; MnP4, manganese porphyrin; MSC, mesenchymal stem cells; MT, matrine; MTC, mannose-modified trimethyl chitosan-cysteine; M6P/IGF IIR, mannose 6-phosphate/insulin-like growth factor II receptor; NCs, nanocrystals; NP, nanoparticle; ODN, oligodeoxynucleotides; PA, CXCR4 ligand; PAI-1, plasminogen activator inhibitor-1; PDGFR, platelet-derived growth factor receptor; PEG, polyethylene glycol; PEG-PLGA, poly(ethylene glycol)-blockpoly(lactide-co-glycolide); PLGA, poly (l-lactide-co-glycolide); PPTT, GNR-mediated photothermal therapy; PRB, probucol; PS, phosphatidylserine; P-SPIONs, pullulan-stabilized iron oxide nanoparticles; RBPR, retinol binding protein receptor; RES, reticuloendothelial system; SeMC, L‑selenium-methylselenocysteine; siRNA, small interfering RNA; SLB, silibinin; SLN, solid lipid nanoparticle; SNALP, stable nucleic acid lipid NPs; SPIONs, superparamagnetic iron oxide; TAA, thioacetamide; Td, tetrahedral DNA; TDN, tetrahedral DNA nanoplatform; TPP, tripolyphosphate; VLNPs, vitamin A-decorated and hyperbranched lipoid-based lipid nanoparticles; LDLR, low-density lipoprotein receptor.

#### 4.2.1 NP-based drug delivery systems targeting HSCs

Given the crucial role of HSCs in hepatic fibrogenesis, targeted NP delivery systems for HSCs have emerged as a focal point in liver fibrosis therapy research (Higashi et al., 2017; Tsuchida and Friedman, 2017). Lipid NP, polymeric NP, inorganic NP, and biomimetic NP-based drug delivery systems modified by HSCs receptor-specific ligands have been employed in liver fibrosis treatment. For instance, PDGFR, mannose 6-phosphate/insulin-like growth factor II receptor (M6P/IGF IIR), C-X-C receptor4 (CXCR4), CD44, and sigma receptor, all of which are abundantly expressed on HSCs surfaces, have been designed with corresponding recognition ligands to deliver antifibrotic drugs to HSCs.

##### 4.2.1.1 Targeting PDGFR

In the process of liver fibrogenesis, PDGF stands out as the most significant mitogen for HSCs. The mitogen PDGF and PDGFR are promising targets for antifibrotic therapies (Wu et al., 2021). Ribera et al. (Ribera et al., 2021a) created gold nanorods coated with anti-PDGFRβ to precisely target activated HSCs *in vivo*. In a CCl_4_-induced mouse model, the photothermal therapy mediated by gold nanorods-PDGFRβ led to a significant decrease in liver fibrosis, inflammation levels, and damage to hepatocytes. Researchers have shown that the cyclic peptide C*SRNLIDC* can act as a targeting ligand for PDGFRβ. Jia et al. (Jia et al., 2018) designed C*SRNLIDC*-liposomes encapsulating siRNA targeting gp46, a collagen-specific chaperone involved in fibrotic deposition. *In vivo* studies revealed that C*SRNLIDC*-liposomes were more effectively taken up by primary mouse HSCs than control liposomes. Within the liver, α-SMA-positive HSCs specifically absorbed C*SRNLIDC*-liposomes, while non-targeted liposomes accumulated in other cell types. In a separate study, Zhang et al. (Zhang et al., 2020) developed pPB peptide (C*SRNLIDC*)-modified HMGB1-siRNA-loaded stable nucleic acid lipid NPs (HMGB1-siRNA@SNALP-pPB). The findings revealed that HMGB1-siRNA@SNALP-pPB were actively targeted to HSCs *via* the mediation of the pPB peptide. This led to the effective silencing of the HMGB1 gene, which subsequently inhibited the activation and proliferation of HSCs, thus reducing collagen deposition and fibrosis formation within the liver.

##### 4.2.1.2 Targeting M6P/IGF IIR

During liver fibrosis, overexpression of M6P/IGF-IIR is detected on the surface of HSCs (Li et al., 2015). Concurrently, M6P-modified HSA (M6P-HSA) demonstrates selectivity toward this receptor, accumulating in activated HSCs within the fibrotic liver. Matrine (MT), an alkaloid derived from *Sophora flavescens*, exhibits diverse bioactivities and has gained clinical application. Recently, MT's antifibrotic properties have been acknowledged, positioning it as a potential inhibitor of liver fibrosis (Wang et al., 2023). Tan et al. (Tan et al., 2023) developed M6P-HSA-MT-SLN, a solid lipid nanoparticle (SLN) encapsulating MT for treating liver fibrosis. The findings indicated that M6P-directed SLNs tend to accumulate preferentially in fibrotic livers and are metabolized slowly. Additionally, M6P-HSA-MT-SLN effectively reduces the markers of liver fibrosis, decreases inflammatory infiltration, and alleviates liver fibrosis.

##### 4.2.1.3 Targeting CXCR4

CXCR4, the predominant chemokine receptor (Wu et al., 2023), is overexpressed in the fibrotic liver and contributes to fibrogenesis by activating HSCs (Hong et al., 2009). AMD3100, when incorporated into NPs, has documented the antifibrotic effects and the ability to recognize HSCs specifically, showcasing the potential of CXCR4 antagonists (Liu et al., 2016). Wu et al. (Wu et al., 2022) constructed a multifunctional CXCR4-inhibitory nano-complex, PA-Zn-CLD/siRNAI-1, comprised of a polymeric AMD3100 (PAMD, PA), Zn^2+^, clodronate (CLD), and siPAI-1 (siRNA of plasminogen activator inhibitor-1). The results illustrate its superior delivery efficiency to HSCs, ultimately serving an antifibrosis function.

##### 4.2.1.4 Targeting CD44

HA, an endogenous glycosaminoglycan biopolymer targeting CD44, has been employed to deliver therapeutic agents to HSCs (Gong et al., 2023). Shinn et al. (Shinn et al., 2024) formulated HA-BR NPs (HABNs), which are composed of endogenous BR, a bile acid known for its antioxidant and anti-inflammatory properties, and HA. The results revealed that HABNs effectively suppress HSCs activation, proliferation, and collagen production. Therefore, in a mouse model of nonalcoholic steatohepatitis (NASH) fibrosis, which was induced by a choline-deficient high-fat diet (CD-HFD), HABNs showed powerful fibrotic regulatory ability. In a separate study, Wang et al. (Wang et al., 2024) developed a multifunctional HA polymeric NP (HA@PRB/COL NPs) incorporating the autophagy inhibitor probucol (PRB) and COL modification. This design enhanced the degradation of ECM and precisely targeted HSCs *via* specificity binding to CD44 receptors, thereby offering a potential therapeutic approach for liver fibrosis. The NPs acted as nano-drills, effectively degrading pericellular collagen I. In a mouse model of liver fibrosis, they specifically targeted HSCs *via* binding to CD44 receptors, resulting in their efficient accumulation in the fibrotic liver. The system offers a potential treatment option for liver fibrosis. Targeting CD44 receptors might be an effective therapeutic strategy for treating liver fibrosis.

##### 4.2.1.5 Targeting integrins αvβ3

Integrins, which are receptors located on the cell surface, exhibit elevated expression levels in some diseased tissues. Each of these compounds includes the arginine-glycine-aspartic sequence (RGD) peptide (Slack et al., 2022). Nanocarriers modified with the RGD peptide have been extensively utilized for targeting HSCs. Notably, Germacrone (GMO) and miR-29b have been demonstrated to significantly inhibit HSCs proliferation and the production of type I collagen (Li et al., 2021; Yu et al., 2017). Ji et al. (Ji et al., 2020) encapsulated GMO and miR-29b in PEG-PLGA-based NPs, which were then functionalized with cyclic RGD peptides (cRGDfK), known as ligands for integrin αvβ3, enhancing their targeting of fibrotic liver tissue (Deepak et al., 2023). The NPs loaded with GMO and miR-29b demonstrated potent cytotoxic effects on activated HSCs and markedly decreased the production of type I collagen. Additionally, cRGD-modified NPs demonstrated remarkable targeting efficiency in liver fibrosis mouse models. These findings suggest that the NPs hold clinical promise for liver fibrosis treatment. While cRGD effectively targets integrins in HSCs, it also interacts with the Type VI Collagen receptor (Du et al., 2007). This could potentially lead to non-specific delivery of cRGD-modified NPs, which might cause their rapid clearance before they extravasate into the liver sinusoid.

##### 4.2.1.6 Targeting RBPR

The primary role of quiescent HSCs is to store fat-soluble vitamin A, also known as retinol. In the serum, vitamin A forms a complex with retinol-binding protein (RBP), which is recognized and internalized by HSCs through the RBP receptor (RBPR). The RBPR is upregulated in activated HSCs. This RBP-vitamin A complex binds to the RBPR, suggesting that it could be a potential target for drug delivery. Kuroda et al. (Kuroda et al., 2015) further confirmed the targeting capability of vitamin A-liposomes in a choline-deficient diet-induced rat liver fibrosis model. These liposomes successfully delivered green fluorescent protein to desmin-positive HSCs in the liver. Qiao et al. (Qiao et al., 2020) developed NPs that target HSCs, grafting them with vitamin A to enable the co-delivery of siCol1α1 and silibinin. This synergistic approach inhibited collagen accumulation, thus alleviating liver fibrosis. The co-delivery of multiple targeted drugs in a combined therapy attained substantial therapeutic outcomes, suggesting a promising new direction for future liver fibrosis treatments.

##### 4.2.1.7 Targeting FGFR1

Fibroblast growth factor 2 (FGF2) primarily exerts its inhibitory effect on HSCs by interacting with FGFR1, highly overexpressed on activated HSCs, and inhibits HSC activation. However, challenges such as a short systemic half-life and low stability, which lead to enzymatic breakdown, restrict the effectiveness of FGF2. Regarding superparamagnetic iron oxide NPs (SPIONs), researchers have proven that they can treat liver fibrosis caused by CCl_4_. A mouse model was used to investigate the impact of SPIONs on CCl_4_-induced hepatic injury. In this model, FGF2 was attached to the SPIONs. *In vitro* research indicated that when TGFβ-activated HSCs were treated with FGF2-SPIONs, the therapeutic potential of FGF2 was significantly enhanced. Additionally, *in vivo* studies revealed a marked increase in efficacy in treating CCl_4_-induced hepatic injury compared to FGF2 alone, which exhibited minimal or no therapeutic effect (Kurniawan et al., 2020). Targeting activated HSCs represents a promising therapeutic approach for liver fibrosis modulation. Existing research has explored multiple targeting strategies using various ligands directed at specific receptors and metabolic pathways in activated HSCs.

#### 4.2.2 NP-based drug delivery systems targeting hepatocytes

The damage to hepatocytes is another important cause of liver fibrosis. Targeting hepatocytes and inhibiting their apoptosis is another important therapeutic strategy for liver fibrosis. NP-based drug delivery systems typically transport hepatoprotective drugs to hepatocytes to preserve liver functionality. Galactose-modified delivery systems can target hepatocytes by interacting with ASGPR, a receptor highly expressed on the surface of hepatocytes (D'Souza and Devarajan, 2015). GalNAc, an amino sugar derivative of galactose, exhibits a higher binding affinity to ASGPR compared to galactose, generating considerable interest in the field of hepatocyte-targeted delivery. Research has demonstrated that the use of GalNAc conjugates to bind to ASGPR for targeted oligonucleotide delivery to liver hepatocytes is a promising strategy in therapeutic oligonucleotide development (Debacker et al., 2020). In addition, Saraswathy et al. (Saraswathy et al., 2021) designed pullulan-stabilized iron oxide nanoparticles (P-SPIONs), a polysaccharide polymer with high specificity for ASGPR, enabling liver-specific diagnostic and therapeutic applications. Selenium (Se), an essential micronutrient, plays a vital role in sustaining human health (Mojadadi et al., 2021). L-*Se*-methyl selenocysteine (SeMC) has demonstrated protective effects against organ injury induced by systemic toxicity related to chemotherapy (Ma et al., 2021). Yuan et al. (Yuan et al., 2023) designed nano-formulated SeMCs, namely GA-SeMC NPs, encapsulated by amphiphilic molecules and glycyrrhetinic acid (GA). The study revealed that GA-SeMC NPs exhibited increased uptake by hepatocytes and greater accumulation in the liver. In APAP- or CCl_4_-induced mouse models, GA-SeMC NPs treatment effectively reduced hepatic lipid peroxidation and serum transaminase levels. Concurrently, it enhanced the antioxidant enzyme expression, demonstrating exceptional hepatoprotective properties.

#### 4.2.3 NP-based drug delivery systems targeting macrophages

NPs have a tendency to amass in the liver and get taken up by hepatic macrophages, which makes them promising options for the treatment of liver fibrosis. A myriad of NP-based drug delivery systems have been developed for target macrophages. Consequently, NPs containing PS are frequently utilized to emulate apoptotic cells, thereby specifically modulating macrophage functions. Wang et al. (Wang et al., 2018) engineered PS-modified nanostructured lipid carriers (mNLCs) encapsulating curcumin (Cur) (Cur-mNLCs) and assessed their therapeutic effectiveness in a rat model of CCl_4_-induced liver fibrosis. The findings revealed that Cur-mNLCs prolonged drug retention *in vivo*, improved bioavailability, and enhanced liver targeting through PS modification. In addition, research has demonstrated that macrophages can be forced into an immunosuppressive and profibrotic M2 phenotype. Notably, M2-type macrophages overexpress the mannose receptor CD206. Kaps et al. designed nanohydrogel particles equipped with mannose residues (ManNP) that delivered colony-stimulating factor 1 receptor (CSF-1R) siRNA more efficiently to M2-polarized macrophages. The results qualified ManNP as promising carriers for siRNA-directed macrophage repolarization in liver fibrosis (Kaps et al., 2020; Leber et al., 2019). Although macrophages can be easily targeted, there is a possibility of being cleared prematurely before they interact with other cells. Therefore, more accurate strategies need to be further explored.

#### 4.2.4 NP-based drug delivery systems targeting other immune cells

Neutrophils, which are innate immune cells in circulation, are drawn to areas of infection and damage. They contribute to hepatic inflammation by producing inflammatory cytokines, activating KCs, and recruiting other immune cells (Xie et al., 2023). Targeting neutrophils to regulate these functions could provide therapeutic benefits. Miettinen et al. (Miettinen et al., 2018) demonstrated that hybrid polymerized liposomal NPs (HPLNs) displaying human and mouse CD177-binding peptides specifically target the CD177-positive neutrophil population. They also identified siRNAs and antisense oligonucleotides as potential tools to knock down C5aR1, a key receptor for neutrophil migration and activation.

Lymphocytes, especially T cells, are capable of selectively eliminating specific antigens and target cells through antigen recognition. The utility of T cells is further enhanced by the approval of cell therapies utilizing genetically modified T cells, namely chimeric antigen receptor (CAR) T cells (Amor et al., 2020). Ramishetti and his co-workers demonstrated that targeted formulations effectively target lymphocytes. They proposed a strategy of coupling anti-CD4 antibodies with LNP (Ramishetti et al., 2015). Subsequently, they showed that β-7 integrin serves as an appropriate ligand for LNP-mediated lymphocyte targeting (Ramishetti et al., 2020). The fact that immune cells take up NPs makes these cells an interesting target for nanomaterial-based therapies.

#### 4.2.5 Nanoparticle-based drug delivery systems targeting LSECs

LSECs play complex and crucial roles in maintaining liver homeostasis. In liver diseases, they are significant factors in promoting inflammation and fibrogenesis. Due to their distinct location, phenotype, and function, LSECs present an attractive target for organ-specific therapies (Shetty et al., 2018). Studies show that LSECs have receptors that recognize and internalize HA, with more than 90 % of circulating HA metabolized by these cells. Thus, HA-modified delivery systems can effectively target LSECs. Ohya et al. (Ohya et al., 2011) formulated polymeric micelles that were conjugated with HA, which was selectively taken up by LSECs. The findings suggest that HA-coated micelles serve as promising drug delivery carriers due to their specific accumulation in LSECs. Yu et al. (Yu et al., 2022) engineered a mannan-targeted, PEGylated PLGA NP (PLGA-PEG-Man) encapsulating Sim, a statin commonly employed for hepatic endothelium protection. The results showed that Sim successfully activated the Kruppel-like factor (KLF2)-NO signaling pathway in LSECs, leading to the reversal of LSEC capillarization and consequently promoting HSCs quiescence. LSEC-targeting NP-based drug delivery systems appear to be promising candidates for the treatment of liver fibrosis. Future therapeutic strategies are likely to increasingly focus on these cells to mitigate liver injury and inflammation, as well as inhibit or reverse fibrogenesis.

### 4.3 Endogenous organ targeting

Beyond ligand-based targeting techniques, the emergence of ligand-free strategies that leverage the endogenous protein corona (PC) demonstrates superior clinical potential. After entering the bloodstream, NPs can attract and adsorb various biomolecules, particularly proteins, onto their surface. This forms a “biomolecular corona” that significantly influences the cellular targeting capabilities of the NPs (Li et al., 2022b). Endogenous organ-targeting LNP delivery to hepatocytes depends on the apolipoprotein E (ApoE)-low-density lipoprotein receptor (LDLR) pathway (Sato et al., 2020). The attachment of the ApoE protein is pivotal for its uptake by the LDLR, which is abundantly expressed in hepatocytes. Kim et al. (Kim et al., 2022) synthesized a DNA tetrahedron that was linked to trivalent cholesterol. This construct exhibited an augmented interaction with serum lipoproteins. This interaction led to the *in situ* formation of lipoprotein-associated protein crowns on the DNA nanostructures, thereby promoting hepatocyte accumulation. In one study, Lin et al. (Liu et al., 2025a) synthesized three types of mannose-modified trimethyl chitosan-cysteine (MTC)-based NPs with distinct surface chemical properties, utilizing tripolyphosphate (TPP), HA, and poly-γ-glutamic acid as crosslinking agents, to encapsulate siRNA *via* ionic gelation. The increased apolipoprotein (APO) B48 levels present within the PC appear to enhance the uptake of orally administered MTC/TPP/siRNA NPs by hepatic macrophages, thereby improving their therapeutic efficacy for acute liver injury. Utilizing the microfluidic device, Younis et al. (Younis et al., 2023b) synthesized a series of LNPs from a diverse library of pH-sensitive lipids. In this process, they identified a promising lipid candidate, CL15A6, exhibiting a high affinity for aHSCs. By adjusting the composition and physicochemical properties of the LNPs, they were able to deliver stable and ligand-free mRNA to aHSCs *in vivo* following intravenous administration. The innovative and scalable platform presented in this study holds significant potential for clinical applications. They also found that CL15A6 and CL15H6 demonstrated high siRNA delivery efficiency to activated HSCs (Younis et al., 2023a). Subsequent in-depth *in vivo* evaluation of these optimized Lipid NPs led to their loading with a combination of two siRNAs targeting SMO and TGFβ1. This approach was designed to concurrently knock down Hh and TGFβ1 signaling pathways, thereby reprogramming activated HSCs into quiescent HSCs and potentially reversing liver fibrosis.

### 4.4 Clinical trials for liver fibrosis

Compared to the extensive range of ongoing preclinical studies, the quantity of clinical trials for nanotherapeutic drugs is notably sparse, and large-scale clinical trials for liver fibrosis remain in their preliminary stages. Based on the current studies, there are no publicly reported FDA/EMA-approved nanoformulations specifically for the treatment of liver fibrosis. Nevertheless, research in this area is progressing rapidly. For instance, in a randomized, placebo-controlled phase II trial, Lawitz et al. (Lawitz et al., 2022) assessed the effectiveness and safety of BMS-986263, a lipid NP formulated to deliver siRNA targeting heat shock protein 47 (HSP47) mRNA degradation, as a potential treatment for advanced fibrosis. The advancements in the pre-clinical phase of NP-based delivery systems have successfully led to the initiation of clinical trials for organic NPs, as presented in Table 3.

**Table 3:** Anti-liver fibrosis nanomedicines in clinical trials.

NPs	Product name	Therapeutic agent	Indications	Phase	Clinical Trial study ID (NCT)
Lipid NPs	ND-L02-s0201	HSP47 siRNA	METAVIR F3–4	Ib/II (2014–2016)	NCT02227459
	BMS-986263	HSP47 siRNA	Advanced liver fibrosis	II (2018–2019)	NCT03420768
Polymeric NPs	BMS-986036	PEG-FGF21	NASH and liver cirrhosis	IIb (2018–2021)	NCT03486912
	SCH 54031	PEG-Intron plus Rebetol	Chronic HCV with liver fibrosis	III (2002–2009)	NCT00049842
	PEG-Interferon Alfa	PEG-Interferon Alfa	HBV-related liver fibrosis	IV (2021–2025)	NCT04640129

Abbreviations: FGF21, fibroblast growth factor 21; HBV, hepatitis B virus; HCV, hepatitis C virus; HSP47, heat shock protein 47; METAVIR F3–4, moderate to extensive liver fibrosis; NASH, nonalcoholic steatohepatitis; PEG, polyethylene glycol.

The challenges faced during the transition from bench to bedside for nanoformulations are as follows: first, the manufacturing of nanoformulations necessitates meticulous control over numerous parameters, including particle size, surface properties, and drug loading efficiency; maintaining consistent quality during large-scale production can be challenging, potentially impacting the efficacy and safety of the final products. Additionally, the absence of distinct guidelines or standards for quality control and characterization of nanomedicines results in inconsistent product quality, potential safety risks, and hurdles in regulatory approval. Furthermore, the safety and biocompatibility of emerging materials have not been thoroughly investigated, which restricts their clinical application. Even when nanoformulations display excellent biocompatibility in pre-clinical studies, they may still trigger an immune response in human trials, thereby posing safety risks. Lastly, the costs associated with preclinical research, clinical trials, and regulatory approval are exceedingly high, potentially impeding the progress of some promising nanoformulation projects (Liu et al., 2025c).

## 5 Conclusions and prospects

Liver fibrosis is an extremely complicated pathological process that is expedited by the interaction of multiple pathogenic mechanisms. Monotherapy with antifibrotic agents is of limited benefit, therefore, to enhance the therapeutic effect, patients must receive significantly elevated dosages, which increases the incidence of side effects. Today, NP-based drug delivery systems are a promising therapeutic tool for various diseases. Nanomedicines, comprised of organic and inorganic nanomaterials, are gaining recognition as innovative therapeutic platforms for *in vivo* systems. Their unique advantages include extended circulation times, reduced off-target cytotoxicity, and enhanced intracellular delivery efficiency. Undoubtedly, nanotechnology is an effective approach for delivering nanomedicine to specific cells in the injured liver. Compared to other organ diseases, the liver environment is more conducive to the entry and retention of nanomaterials, offering inherent advantages for NP applications.

In the absence of a targeting moiety, all NPs invariably accumulate in the liver, which consequently raises concerns about their potential hepatotoxicity. Furthermore, due to altered hemodynamics, ECM mechanical properties, and complex physiological processes in liver fibrosis, NPs encounter challenges in reaching fibrotic liver tissue. Designing NPs with an appropriate size and shape can improve their ability to penetrate the non-fenestrated sinusoidal endothelium and the stroma-rich microenvironment. Modifying the surface of NPs with specific ligands or polymers can also enhance their penetration. Active targeting strategies have high specificity, which can accurately identify and combine, thereby improving the enrichment of NPs at the target site. However, the expression of receptors *in vivo* may be affected by various factors, such as fibrotic ECM, possibly interfering with ligand-receptor binding, and long-term use may lead to receptor downregulation or immune responses, affecting targeting effects. In addition, the screening and modification process of ligands is relatively complex and costly.

There are many NPs in preclinical trials, however, only a few are approved and tested in clinical trials for liver fibrosis. To obtain more innovative, safe, and effective NP delivery systems, deeper cooperation in the fields of materials science, basic medicine, and biochemistry is needed to create more ingenious NPs. In addition to nanomedicines, several emerging technologies are gaining traction and competing for attention and funding that was once primarily directed toward nanomedicine. These novel approaches include smart stimuli-responsive NPs, theranostics (Muthu et al., 2014), AI-driven NP design, or organoid-based screening platforms (Gerbolés et al., 2023). The integration between nanomedicines and emergent technologies will shape the future path of molecular medicine in the forthcoming years.

## CRediT authorship contribution statement

**Ya-Ning Chen:** Writing – original draft. **Meng-Qi Li:** Software, Resources. **Hui-Juan Zhang:** Software, Resources. **Na-Na Xu:** Investigation. **Yu-Qian Xu:** Investigation. **Wen-Xuan Liu:** Investigation. **Ting-Ting Chen:** Visualization. **Nan Li:** Visualization. **Guang-Yang Wu:** Writing – review & editing. **Jie-Min Zhao:** Writing – review & editing, Supervision. **Wu-Yi Sun:** Writing – review & editing, Supervision, Funding acquisition.

## Declaration of competing interest

The authors declare the following financial interests/personal relationships which may be considered as potential competing interests:

## Data Availability

No data was used for the research described in the article.
